# Obese Mice with Dyslipidemia Exhibit Meibomian Gland Hypertrophy and Alterations in Meibum Composition and Aqueous Tear Production

**DOI:** 10.3390/ijms21228772

**Published:** 2020-11-20

**Authors:** Eugene A. Osae, Tiffany Bullock, Madhavi Chintapalati, Susanne Brodesser, Samuel Hanlon, Rachel Redfern, Philipp Steven, C. Wayne Smith, Rolando E. Rumbaut, Alan R. Burns

**Affiliations:** 1 College of Optometry, University of Houston, Houston, TX 77204, USA; sdhanlon@gmail.com (S.H.); rredfer2@central.uh.edu (R.R.); arburns2@central.uh.edu (A.R.B.); 2 Children’s Nutrition Research Center, Baylor College of Medicine, Houston, TX 77030, USA; tbullock@bcm.edu (T.B.); chintala@bcm.edu (M.C.); cwsmith@bcm.edu (C.W.S.); rrumbaut@bcm.edu (R.E.R.); 3 CECAD Research Center, Lipidomics/Metabolomics Facility, University of Cologne, 50931 Cologne, Germany; susanne.brodesser@uk-koeln.de; 4 Department of Ophthalmology, Division for Dry-Eye and Ocular GvHD, Medical Faculty, University of Cologne, 50937 Cologne, Germany; philipp.steven@uk-koeln.de; 5 Center for Translational Research on Inflammatory Diseases (CTRID), Michael E. DeBakey Veterans Affairs Medical Center, Houston, TX 77030, USA

**Keywords:** obesity, dyslipidemia, meibum, lipids, meibomian gland dysfunction, dry eye, mass spectrometry

## Abstract

Background: Dyslipidemia may be linked to meibomian gland dysfunction (MGD) and altered meibum lipid composition. The purpose was to determine if plasma and meibum cholesteryl esters (CE), triglycerides (TG), ceramides (Cer) and sphingomyelins (SM) change in a mouse model of diet-induced obesity where mice develop dyslipidemia. Methods: Male C57/BL6 mice (8/group, age = 6 wks) were fed a normal (ND; 15% kcal fat) or an obesogenic high-fat diet (HFD; 42% kcal fat) for 10 wks. Tear production was measured and meibography was performed. Body and epididymal adipose tissue (eAT) weights were determined. Nano-ESI-MS/MS and LC-ESI-MS/MS were used to detect CE, TG, Cer and SM species. Data were analyzed by principal component analysis, Pearson’s correlation and unpaired *t*-tests adjusted for multiple comparisons; significance set at *p* ≤ 0.05. Results: Compared to ND mice, HFD mice gained more weight and showed heavier eAT and dyslipidemia with higher levels of plasma CE, TG, Cer and SM. HFD mice had hypertrophic meibomian glands, increased levels of lipid species acylated by saturated fatty acids in plasma and meibum and excessive tear production. Conclusions: The majority of meibum lipid species with saturated fatty acids increased with HFD feeding with evidence of meibomian gland hypertrophy and excessive tearing. The dyslipidemia is associated with altered meibum composition, a key feature of MGD.

## 1. Introduction

Obesity has reached epidemic levels, affecting about 2 billion adults and 42 million children globally, and the associated complications, including type 2 diabetes, are on the rise [1,2,3,4,5]. In the United States alone, the obesity epidemic has been linked to various chronic diseases with high mortality and financial burden. It is estimated to result in about 300,000 deaths and over USD 100 billion in direct and indirect costs annually [1,2,3,4,5].

As a major component of the metabolic syndrome, obesity is often accompanied by dyslipidemia (abnormal blood lipid profile) [4,6,7,8]. Most dyslipidemias are hyperlipidemias, characterized by elevated (atherogenic) levels of blood lipids, such as cholesterol, triglycerides and low-density lipoproteins, and/or a reduction in high density lipoproteins. Dyslipidemia is a well-known risk factor for heart disease [8,9,10].

In the eye, dyslipidemia is also thought to initiate meibomian gland dysfunction (MGD), the leading cause of dry eye disease [11,12]. The meibomian glands are modified sebaceous glands in the eyelids which produce meibum, the tear film lipid component [13]. Meibum is critical for ocular surface homeostasis [14]. It stabilizes and aids in the spreading of the tear film and retards tear film evaporation, thereby preventing dry eyes and ocular surface damage [13,14]. Meibum is also thought to have antimicrobial properties that prevent infection at the ocular surface, suggesting MGD could be detrimental to ocular surface health [13,15].

According to a report by Butovich et al., the composition of meibum in humans is similar to other species, including the mouse. Human and mouse meibum are similar in percentage composition of various lipids, including wax esters, cholesterol, cholesteryl esters, diglycerides, triglycerides, O-acyl-ω-hydroxy fatty acids and sphingolipids [16,17,18,19,20]. The majority of these lipid classes, among others, are also found in blood [21,22]. Since the meibomian glands produce these lipids that constitute meibum, and blood and meibum profiles share some commonalities, several anecdotal reports suggest that dyslipidemia, as occurs in obesity, can contribute to MGD, including alterations in meibum lipid composition. Disruption of the normal lipid composition could also be pro-inflammatory, serving as a potential precursor for blepharitis, which can affect the normal secretory function of the meibomian gland [13,23,24]. This can lead to reduced tear film quality and stability and, ultimately, ocular surface inflammation as occurs in MGD and dry eye [4,11,12,13,16,17,18].

Over the last decade, the idea has gained momentum that blood plasma lipids affect the meibomian gland [12,13,14,17]; however, direct experimental evidence for this relationship is lacking. Moreover, one experimental study dating back to 1975 was unable to confirm this relationship [25]. Hence, there is a need to re-examine this relationship using an appropriate experimental approach that includes a suitable animal model and sensitive analytical techniques [12,13]. 

Herein, we employ meibography to assess meibomian gland structural changes in a diet-induced obesity mouse model where mice develop dyslipidemia [12,26,27]. Most importantly, and for the first time, nano-electrospray ionization tandem mass spectrometry (Nano-ESI-MS/MS) and liquid chromatography coupled with electrospray ionization tandem mass spectrometry (LC-ESI-MS/MS) are used to provide a detailed and sensitive analysis of the relational status of blood (plasma) lipids and meibum lipids. Functional consequences on tear production are also discussed.

## 2. Results

### 2.1. Mice Fed an Obesogenic Diet Gained More Weight with Elevated Total Plasma Lipid Levels

**Figure 1:**
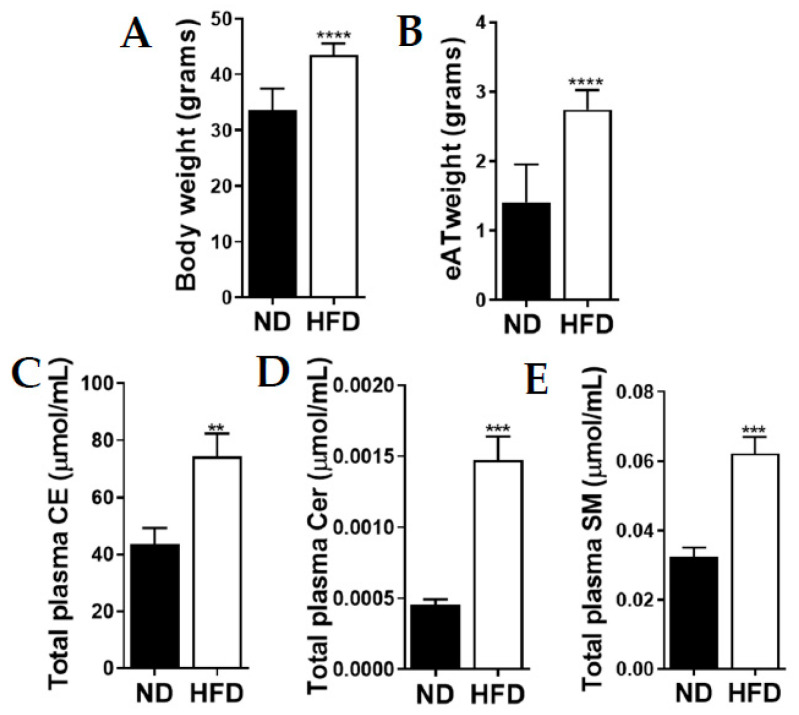
High-fat diet (HFD)-fed mice (**A**) weighed significantly more and showed (**B**) heavier epididymal adipose tissue (eAT) than normal diet (ND)-fed mice. HFD mice also showed elevated total fasting plasma (**C**) cholesteryl ester (CE), (**D**) ceramide (Cer) and (**E**) sphingomyelin (SM) levels. Mouse weight and eAT are expressed as means ± SD and all total lipid data are expressed as means ± SEM. ** *p* < 0.01, *** *p* < 0.001, **** *p* < 0.0001. Asterisks indicate a significant difference compared to the matched ND value.

At the end of the feeding period, HFD mice weighed 30% more than ND mice, *p* < 0.0001. The HFD mice also showed greater (2-fold) eAT weight than the ND mice, *p* < 0.0001 (Figure 1A,B). These changes were accompanied by higher (total fasting) levels of the various plasma lipids detected. The average (total fasting) plasma CE, Cer and SM levels were ≈ 2 to 3-fold higher in the HFD mice compared to the ND mice, *p* < 0.01–<0.001 (Figure 1C–E). Total levels of plasma TG-18:2, TG-18:3, TG-20:4 and TG-22:6 were, however, ≈3 to 4 fold lower in the HFD mice compared to the ND mice, *p* < 0.0001 (see Appendix C).

### 2.2. Relationship between Mouse Body Weight, eAT and Total Fasting Plasma Lipid Levels

**Figure 2:**
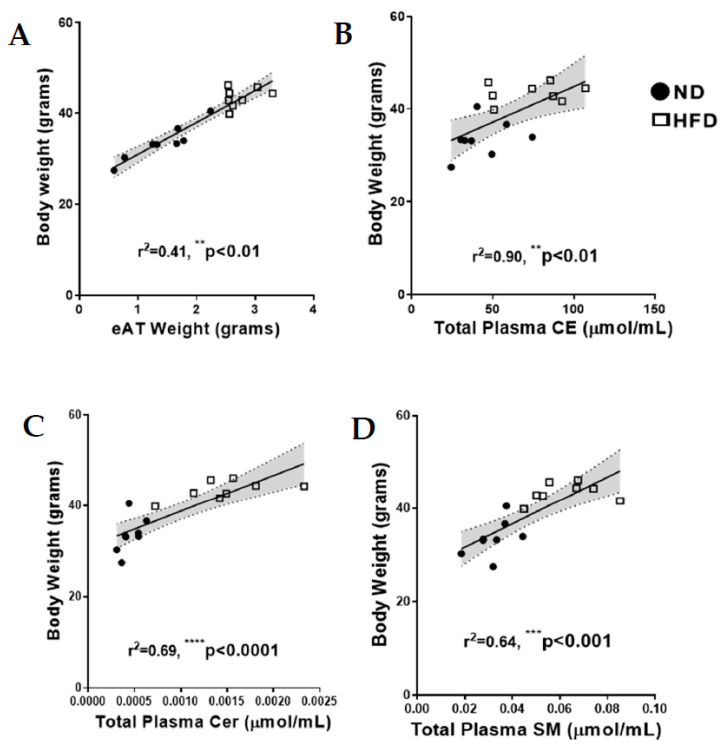
Correlation between mouse body weight and (**A**) epididymal adipose tissue (eAT) and total plasma (**B**) cholesteryl esters (CE), (**C**) ceramides (Cer), and (**D**) sphingomyelins (SM). The grey-shaded regions represent the 95% confidence interval. Asterisks indicate a significant difference compared to the matched ND value.

A strong positive and significant correlation was observed between mouse body weight and eAT. Similarly, mouse body weight correlated strongly and significantly with the (total fasting) levels of plasma CE, Cer and SM (Figure 2A–D). In contrast, significant negative correlations were observed between mouse body weight and total levels of plasma TG-18:2, TG-18:3, TG-20:4 and TG-22:6 (see Appendix D).

### 2.3. Mice Fed an Obesogenic Diet Showed Meibomian Gland Hypertrophy and Excessive Tear Production

**Figure 3:**
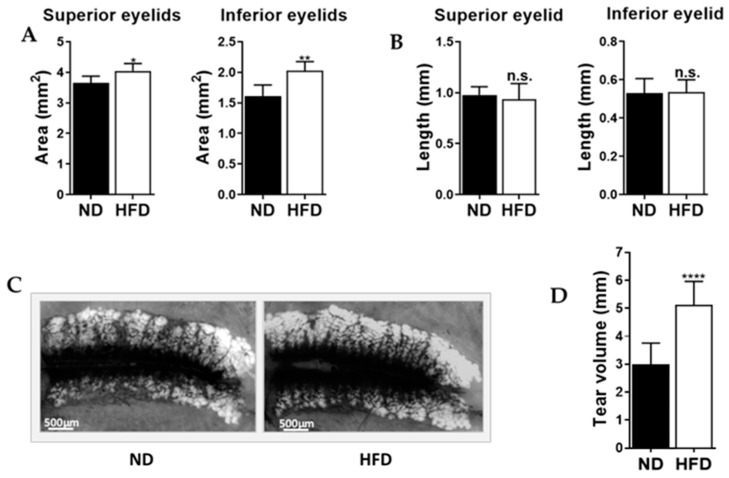
HFD mice showed (**A**) larger meibomian gland area and (**B**) similar meibomian gland length. (**C**) Representative meibographs showing a typical set of glands from an ND mouse and an HFD mouse. (**D**) HFD mice also showed greater tear volume than ND mice. All data are expressed as means ±SD. * *p* < 0.05, ** *p* < 0.01, **** *p* < 0.0001, n.s. = not significant. Asterisks indicate a significant difference compared to the matched ND value.

Compared to the ND mice, the HFD mice showed larger meibomian gland area in both the superior (*p* < 0.05) and inferior eyelids (*p* < 0.01). Meibomian gland length, however, did not differ significantly between the groups. Tear volume was significantly greater in the HFD mice than the ND mice, *p* < 0.0001, (Figure 3A–D).

### 2.4. Similarities and Differences between Plasma and Meibum Lipid Species

In all four lipid classes studied, a total of 27 different lipid species were detected, based on fatty acid moiety classification. Out of these, we detected 19 species in plasma and 25 in meibum, with 17 species being shared. Unlike plasma, meibum contained some odd-chained fatty acid species (C15:0, 17:0, 19:0, 21:0 and 25:0). The known common names, chemical formula and melting points of the fatty acids of the detected lipid species [28] are listed in Table 1.

### 2.5. Relative Abudance of the Lipid Species within the Various Lipid Classes in Plasma and Meibum

#### 2.5.1. Cholesteryl Esters 

**Figure 4:**
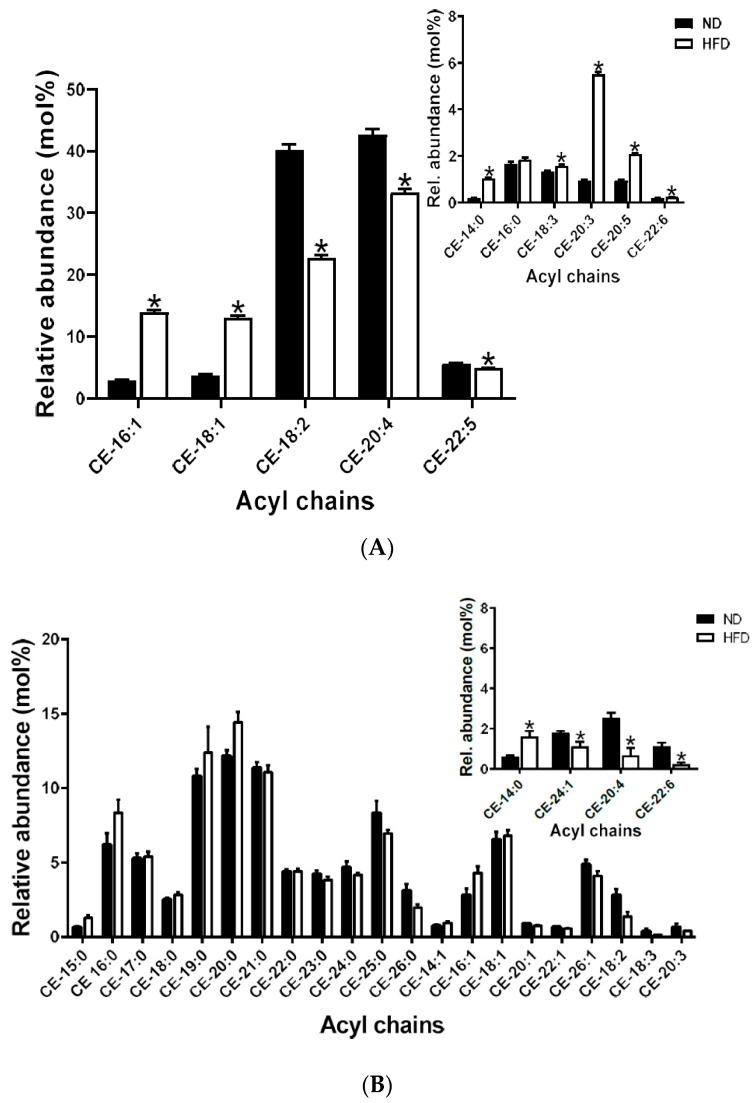
(**A**) Plasma cholesteryl ester species detected; inset shows species with low relative abundance. Data are expressed as means ± SEM, * *p* < 0.05. Asterisks indicate a significant difference compared to the matched ND value. (**B**) Meibum cholesteryl ester species detected; inset shows species with low relative abundance. Data are expressed as means ± SEM, * *p* < 0.05. Asterisks indicate a significant difference compared to the matched ND value.

CE-20:4 was the most abundant species in plasma. This was followed by CE-18:2, and the least abundant species was CE-14:0. General increases in the saturated, monounsaturated (MUFA) and polyunsaturated (PUFA) species were observed after HFD feeding, *p* < 0.05, (Figure 4A).

Compared to plasma, meibum CE sub-lipidome showed a broader array of species. The most abundant species was CE-20:0, followed by CE-19:0 and CE-21:0 with CE-18:3 being the least abundant. We observed an increase in certain saturated species (CE:14:0) and MUFA species (CE-24:1) and decreases in some PUFA species (CE-20:4 and CE-22:6), *p* < 0.05, (Figure 4B).

#### 2.5.2. Triglycerides

**Figure 5:**
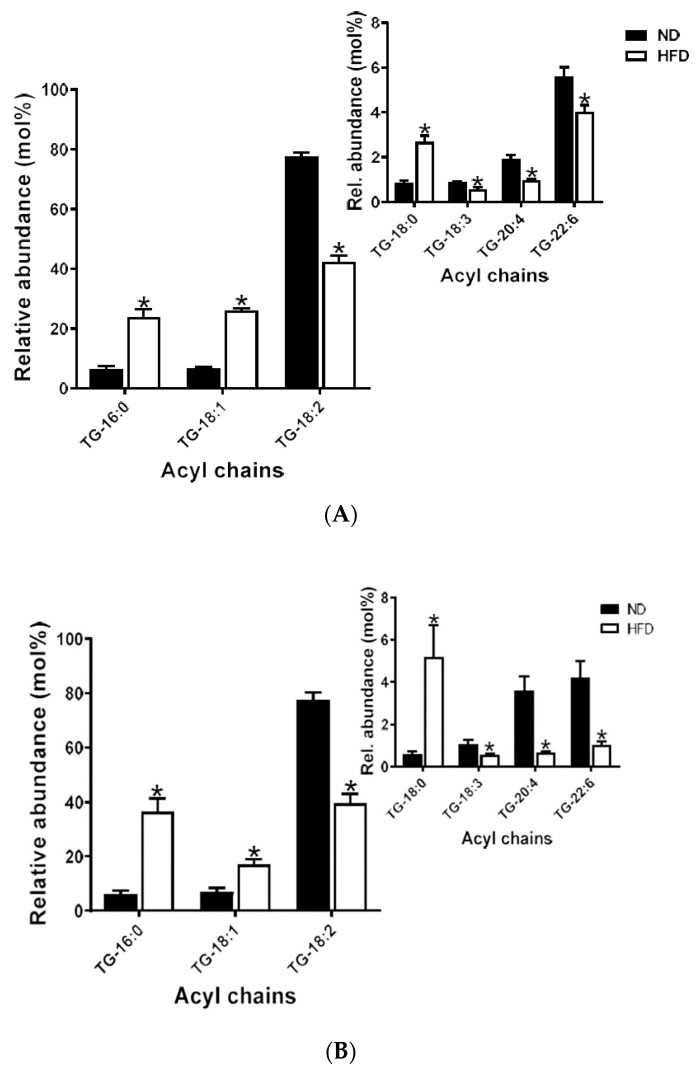
(**A**) Plasma triglyceride species detected; inset shows species with low relative abundance. Data are expressed as means ± SEM, * *p* < 0.05. Asterisks indicate a significant difference compared to the matched ND value. (**B**) Meibum triglyceride species detected; inset shows species with low relative abundance. Data are expressed as means ± SEM, * *p* < 0.05. Asterisks indicate a significant difference compared to the matched ND value.

In plasma, TG-18:2 was the most abundant, and TG-18:3 was the least. There were significant increases in all saturated (TG-16:0 and TG-18:0) and MUFA (TG-18:1) species detected in plasma after HFD feeding. On the other hand, all PUFAs were significantly decreased after HFD feeding, (Figure 5A). A similar pattern of increases in all saturated and MUFA species and decreases in PUFAs after HFD feeding was also observed in meibum, *p* < 0.05, (Figure 5B).

#### 2.5.3. Sphingomyelins

**Figure 6:**
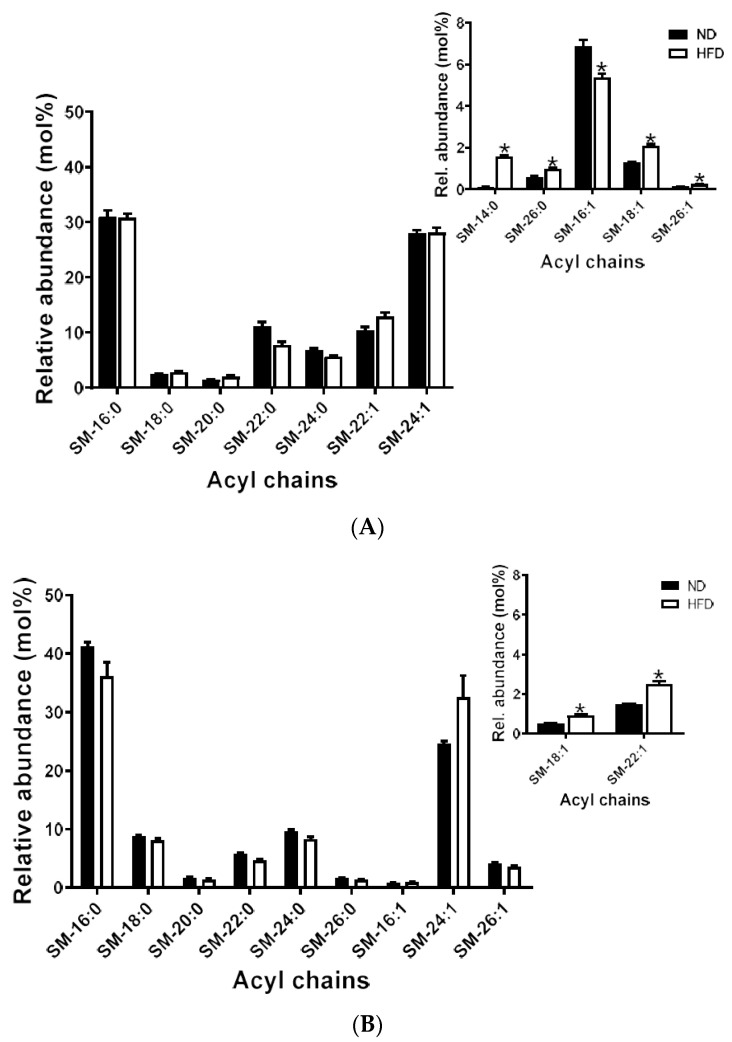
(**A**) Plasma sphingomyelin (SM) species detected; inset shows species with low relative abundance. Data are expressed as means ± SEM, * *p* < 0.05. Asterisks indicate a significant difference compared to the matched ND value. (**B**) Meibum sphingomyelin species detected; inset shows species with low relative abundance. Data are expressed as means ± SEM, * *p* < 0.05. Asterisks indicate a significant difference compared to the matched ND value.

Unlike cholesteryl esters and triglycerides, only saturated and MUFA sphingomyelin species were detected in both plasma and meibum. In plasma, SM-16:0 was the most abundant species, followed by SM-24:1, with SM-26:1 being the least detected species. Some saturated (SM-14:0 and SM-26:0) and MUFA (SM-16:1, SM-18:1 and SM-26:1) species were significantly elevated after HFD feeding; Figure 6A. In meibum, SM-16:0 was the most abundant and SM-16:1 was the least abundant species. SM-18:1 and SM-22:1 were significantly elevated after HFD feeding; Figure 6B.

#### 2.5.4. Ceramides

**Figure 7:**
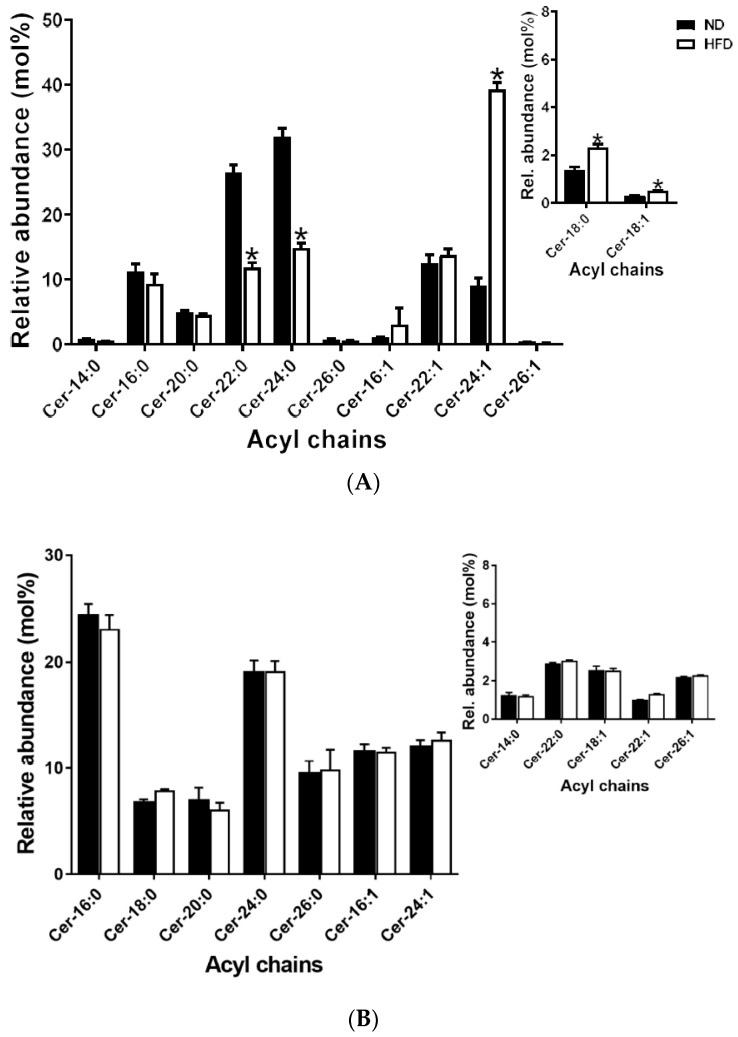
(**A**) Plasma ceramide species detected; inset shows species with low relative abundance. Data are expressed as means ± SEM, * *p* < 0.05. Asterisks indicate a significant difference compared to the matched ND value. (**B**) Meibum ceramide (Cer) species detected; inset shows species with low relative abundance. Data are expressed as means ± SEM and no significant difference was recorded.

Similar to the sphingomyelins, only saturated and MUFA species were detected in Cer in both plasma and meibum. In plasma, Cer-18:0, Cer-18:1 and Cer-24:1 were significantly elevated after HFD feeding, whereas Cer-22:0 and Cer-24:0 were significantly decreased after HFD feeding. Meibum Cer species did not change after HFD feeding; Figure 7A,B.

### 2.6. Patterns of Variation in the Different the Lipid Species within the Vairous Lipid Classes in Plasma and Meibum

#### 2.6.1. Cholesteryl Esters

**Figure 8:**
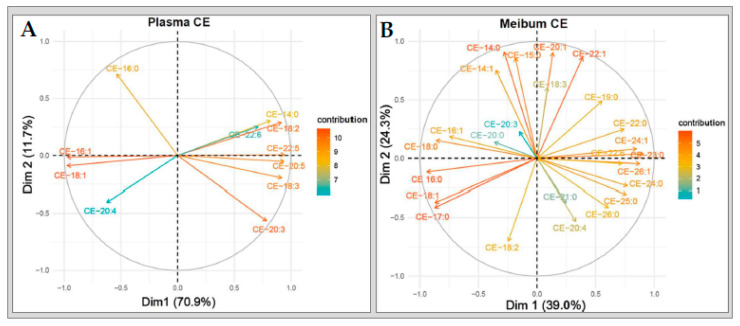
Variable correlation plots of (**A**) plasma CE and (**B**) meibum CE species.

The principal component analyses showed that plasma CE species tend to change together in a particular direction based on their unsaturation characteristics, that is, whether they are saturated, MUFA or PUFA types. Overall, the change in plasma CE PUFA species was the opposite of that of CE MUFA and saturated species. In meibum, however, this directional change occurred based on acyl chain lengths. Long-chain meibum CE species appeared to change collectively in a manner that is opposite to very-long-chain meibum CE species. In addition to these, we observed that long-chain MUFAs contributed the most to the overall variance in the plasma CE sub-lipidome while long- and very-long-chain PUFAs contributed the least. In contrast to plasma, the greatest contributors to the overall variance in meibum CE sub-lipidome resulted from a mix of long- and very-long-chain saturated or MUFA types, (Figure 8A,B).

#### 2.6.2. Triglycerides

Another important observation was that all triglyceride PUFA species tend to change together in a common direction, in both plasma and meibum. This direction of change is inversely related to that of the saturated and MUFA species. In plasma, the greatest contributor to the overall variance in the in the TG sub-lipidome resulted from long-chain saturated PUFA species (TG-18:3 and TG 20:4) and the least from only one very-long-chain PUFA species (TG-22:6).

**Figure 9:**
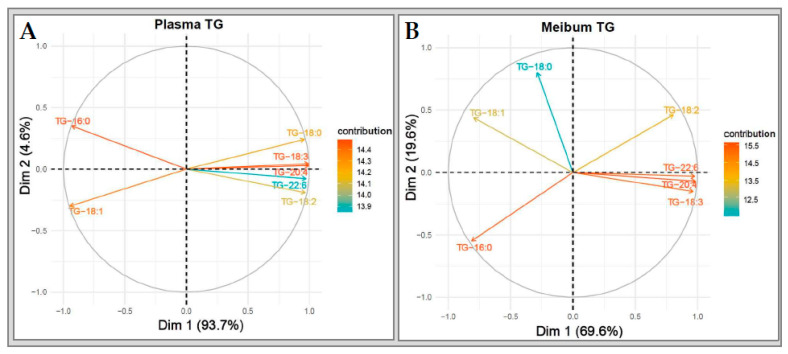
Variable correlation plots of (**A**) plasma TG and (**B**) meibum TG species.

In contrast, the greatest contributions to the overall variance in meibum TG sub-lipidome were mainly due to both long- and very-long-chain PUFAs, with the least contributions from long-chain saturated species (TG-18:0) (Figure 9A,B). 

#### 2.6.3. Sphingomyelins and Ceramides

**Figure 10:**
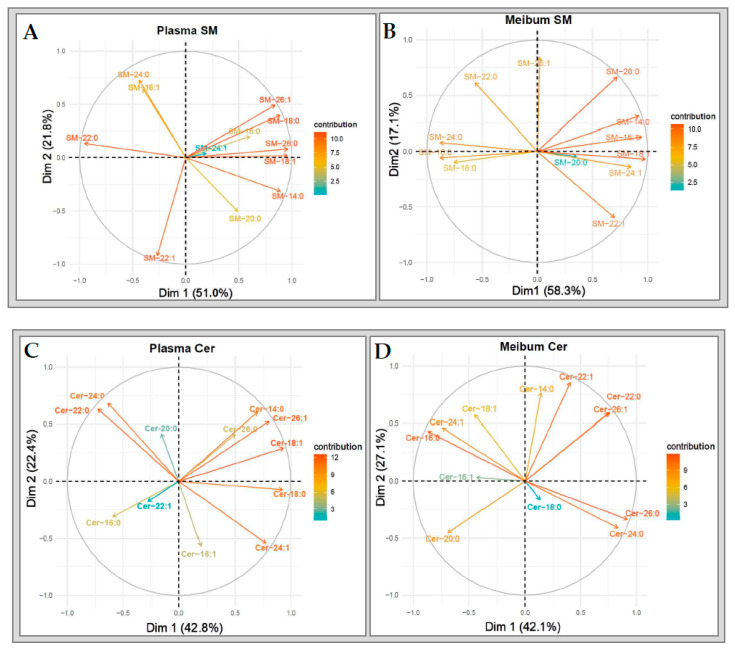
Variable correlation plots of (**A**) plasma SM and (**B**) meibum SM species. Variable correlation plots of plasma (**C**) Cer and (**D**) meibum Cer species.

Unlike CE and TG, the patterns of change in the sphingolipids (SM and Cer) were diffuse in both plasma and meibum. Furthermore, the contribution levels of the various species to the overall variances in both SM and Cer sub-lipidomes did not conform to unsaturation characteristics or acyl chain lengths (Figure 10A–D).

## 3. Discussion

In this study, our objectives were to assess meibomian gland structural changes and to evaluate and present first-time data on the relational status of blood (plasma) lipids and meibum lipids in a diet-induced obesity model where mice develop dyslipidemia. 

The obesogenic diet used approximates the contemporary (Western) diet compositions, and as expected, the HFD mice gained more weight, which is accompanied by significant elevations in (fasting) plasma lipid levels, indicative of dyslipidemia, specifically hyperlipidemia [6,29,30]. The increase in mouse body weight correlates with increases in epididymal adipose tissue, indicating increased adiposity [6,31]. Furthermore, mouse body weight also correlates with the increased (fasting) levels of the various lipid classes common to obesity and the metabolic syndrome [6,32,33].

In examining the eyelids, we observed that HFD mice present significantly larger meibomian gland areas in both the upper and lower lids compared to ND mice, which other studies suggest may be attributable to apparent widening of the glands [13,34]. Interestingly, there were no differences in gland length between the groups. Widening of the meibomian gland is thought to precede meibomian gland dysfunction and exacerbate ocular surface disease [13,34,35,36]. Similar findings have been reported in other studies [34,36]. However, these studies attribute the hypertrophic changes in the meibomian glands to desiccating stress, allergy, inflammation-mediated events and hyperkeratinization of the meibomian gland [13,34,35,36]. Therefore, this makes our work the first to document and report such hypertrophic changes in a model where mice exhibit dyslipidemia.

It is not fully understood how an obesogenic diet and the resulting dyslipidemia can affect the meibomian gland secretion [12,13,25]. However, there is evidence that dietary factors can alter normal functioning of sebaceous glands [37]. For example, sebum production by skin sebaceous glands has been shown to be altered in volume and composition by the increased consumption of dietary fat or carbohydrate [38,39]. Furthermore, dietary restriction has been shown to reduce sebum secretion rate in the skin [40,41]. The understanding is that these diets supply substrates for sebum synthesis. Therefore, the meibomian gland being a modified sebaceous gland is likely affected by the obesogenic diet [11,12,13]. Moreover, our previously published evidence of vasculature around the meibomian gland suggests the possibility of uptake of dietary substrates from the blood stream for meibum lipid synthesis—a process which may be altered during dyslipidemia [11,12,13]. 

To understand how a dyslipidemic status resulting from an obesogenic diet can contribute to lipid compositional changes, we targeted plasma and meibum CE, TG, Cer and SM [16,17,18,42]. This is because these lipids are present in both blood plasma and meibum. In meibum, these lipids fall into two broad classes: non-polar (CE and TG) and polar (Cer and SM) [16,17,18,42]. The non-polar lipids occupy the air–tear film lipid layer interface, whereas the polar and more amphiphilic lipids occupy the tear film–mucin–aqueous interface of the tear film. 

Non-polar lipids are especially thought to prevent evaporation, provide a clear and smooth corneal refraction surface and serve as external barrier against foreign bodies. The polar lipids, on the other hand, enable the interaction between the outermost non-polar lipids and the inner aqueous tear film component [14,42]. Polar lipids further ensure tear film stability by lowering surface tension and increasing the viscoelastic properties of the aqueous tear film component [14,42]. This is very important for proper separation of the various tear film molecules, spreading of the tear film and prevention of ocular surface dryness [14,42]. The attempt to study these lipid classes enables us to understand how dyslipidemia induced by an obesogenic diet can affect both polar and non-polar meibum lipids.

It has also been widely reported that elevated levels of these lipid classes in plasma can result in significant morbidity and mortality [8,9,43,44]. However, the implications of such lipid alterations in the meibomian gland, especially during dyslipidemia, have not been fully explored [13,16,17,18]. In employing Nano-ESI-MS/MS and LC-ESI-MS/MS to analyze the samples, the lipid species detected in this study are similar to what Butovich et al. reported. In terms of fatty acid moieties, we detected species with C_14_ to C_26._ These are suggested to be either major precursors or metabolic products of meibogenesis [20]. Fatty acid moieties with C_24_ or longer may possibly exhibit surfactant properties, a feature of lipids that is critical for tear film stability and spread at the ocular surface [14,20].

Most importantly, a general decrease in unsaturation (i.e., increased saturation) of both plasma and meibum CE, TG and SM species in the HFD mice was observed. Alterations in Cer species occurred only in plasma and not meibum. We further observed well-defined variability in the non-polar lipid (CE and TG) species in both plasma and meibum, based on either unsaturation characteristics or acyl length. This type of variability was however not well-defined in the polar lipids (SM and Cer). The extent and variability of alteration may depend on the saturation, acyl chain length and /or polarity of the lipids as demonstrated by the principal component anlayses [16,17,18].

In meibum, the observed general increases in saturated lipid species in the HFD group can have several consequences at the ocular surface (Figure 4B, Figure 5B and Figure 6B). For example, increased meibum lipid saturation is reported to cause stiffness of meibum lipids, causing the tear film lipid layer or component to interact poorly with other tear film components, such as aqueous or mucin proteins, thus resulting in tear film instability and impaired spreading at the ocular surface. This can, in turn, lead to dry eye and ocular surface damage. In fact, existing reports suggest that meibum from donors with meibomian gland dysfunction demonstrates elevated levels of acyl chain saturation of constituent lipids [45,46,47]. 

Increased lipid saturation has been shown to have a strong impact on the surface properties of meibum, including impaired heterogeneity in the structure of the various meibum layers (polar and non-polar) and increased acyl chain order and acyl chain-melting phase transition temperature. The latter effect is due to the fact that saturated lipids lack double bonds, and so they pack tightly under strong van der Waals forces [13,47]. As demonstrated in Table 1, saturated lipid species have higher melting points compared to unsaturated types. This, together with longer acyl chain length in meibum lipids, can result in potential qualitative changes in meibum, such as high bulk viscosity—and in humans, this is reported to result in impaired flow of meibum from the meibomian gland [48].

That is, increased saturation and elongation of the various lipid species can render meibum less fluid and more “toothpaste-like”, as is often the case for patients with meibomian gland dysfunction. The increased viscosity can lead to stasis of meibum in the gland which may explain the dilation of the glands that we observed after HFD feeding [13,46,47,48]. Such a reduction in meibum flow to the ocular surface can also elicit compensatory tear aqueous and mucin component production, leading to watery eyes—a common clinical sign in some patients with dry eye and meibomian gland dysfunction [13,49]. Interestingly, we realized that the HFD mice showed watery eyes and this was confirmed by tear volume readings from our phenol red thread test. This finding is similar to that of one other study where knockout mice with impaired lipid metabolism showed compensatory increases in the tear aqueous and mucin components as a result of defective meibomian glands [49].

Furthermore, since dyslipidemia exists in a complex relationship with inflammation, the obesogenic diet-induced dyslipidemia and the alterations in meibum lipid composition in our model can lead to possible inflammatory events in the meibomian gland. This can lead to MGD and ocular surface inflammation. While there is lack of consensus on the involvement of inflammation in MGD, previous studies have reported that a high-fat diet can lead to increased oxidative stress and inflammatory cell and cytokine infiltration around the acini of the mouse lacrimal gland acini [50]. In fact, we recently published that mice fed the obesogenic also show increased levels of systemic and corneal inflammatory mediators [51], and considering the ocular surface as functional unit, it is possible that the diet-induced dyslipidemia and the alterations in meibum lipids can be pro-inflammatory, impacting the meibomian gland in a similar fashion [13]. This can contribute to MGD and perpetuate the vicious circle of MGD and dry eye [13].

In summary, this study supports the idea that an obesogenic diet not only results in dyslipidemia but also alterations in meibomian gland structure (hypertrophy) and function (saturation in meibum lipids), which are associated with compensatory (excess) aqueous tear production features consistent with dry eye and MGD [13,49]. These findings underscore diet as important factor that may be worth considering as part of the therapeutic strategies when managing MGD.

## 4. Methods

### 4.1. Ethical Cosniderations

The experimental protocols for this study were approved by the Baylor College of Medicine (AN-2721; 24-03-2020) and the University of Houston (UH16-005; 16-03-2020) Institutional Animal Care and Use policies. The animals were cared for and treated in accordance with the institutional ethical guidelines. All procedures were performed according to the Association for Research in Vision and Ophthalmology (ARVO) Statement for the Use of Animals in Ophthalmic and Vision Research.

### 4.2. Mouse Model, Tear Production Assessments, Meibography and Sample Collection

Male C57/BL6 mice (n = 8/group; 5-week-old) were fed a normal (ND; 15% kcal fat) or an obesogenic high-fat diet (HFD; 42% kcal milk fat, diet#112734, Dyet Inc., Bethlehem, PA, USA) for 10 weeks. The decision for a 10-week feeding period was premised on our previous consistent findings of pathological corneal changes in the model at 10 weeks and the consideration of the ocular surface as a single functional unit where pathological changes seen in any of its components (e.g., the cornea changes may likely occur simultaneously with pathology of the other components)—in this case, the meibomian gland [26,52,53]. At the end of the feeding period, mice were fasted for 5 h. Prior to deep anesthesia using 2% isoflurane and subsequent weighing of fasted mice, tear production was measured using the phenol red thread test, as already described [34]. The mice were then euthanized by cervical dislocation. Blood was immediately collected by cardiac puncture using (10 U/L) heparinized syringes into 1.5-mL Eppendorf tubes and then centrifuged for 15 min (3000 rpm, 4 °C) to obtain plasma. Mouse epididymal adipose tissue (eAT) was collected and weighed. The eAT is a piece of fat that sits on the testes in male vertebrates and serves as a useful biomarker for visceral adiposity in mice [6]. Eyelids were carefully excised and meibography immediately performed as previously described [34]. Using platinum spatula and forceps, meibum was collected by careful manual expression of the eyelids into 3:1 (*vol*/*vol*) chloroform: methanol organic solvent in 1 Dram amber glass vials with Teflon-lined caps [20]. This was to prevent leaching and ensure the integrity of meibum samples. The organic solvent was evaporated and dry meibum samples were weighed and stored with the plasma samples at −80 °C until lipidomic analyses [27].

### 4.3. Lipidomic Analyses of Plasma and Meibum Samples

Lipidomics analyses of plasma and meibum samples were done at the Lipidomics/Metabolomics Core Facility of the Cluster of Excellence: Cellular Stress Responses in Aging-Associated Diseases (CECAD) at the University of Cologne, Germany. Targeted analyses of plasma and meibum cholesteryl esters, triglycerides, sphingomyelins and ceramides were performed.

#### 4.3.1. Analysis of Cholesteryl Ester and Triglycerides Species

Cholesteryl ester and triglyceride species were quantified by nano-electrospray ionization tandem mass spectrometry (Nano-ESI-MS/MS). To dry meibum and 5 μL of plasma, respectively, 500 µL of Milli-Q-water, 1.875 mL of chloroform/methanol/37% hydrochloric acid 5:10:0.15 (*v*/*v*/*v*) and internal standards (IS) were added. For the plasma samples, 15 μL of 4 μM d5-TG Internal Standard Mixture I and 20 µL of 256 µM cholesteryl ester 19:0 from Avanti Polar Lipids (Alabaster, USA) were used as internal standards. For the meibum samples, 20 µL of the 4 μM d5-TG mixture was used. Since cholesteryl ester 19:0 is endogenously present in mouse meibum, we refrained from adding any internal standard for cholesteryl esters to the meibum samples [54]. Lipids were extracted using the “One-Step Extraction” described by Özbalci et al. [27], a method modified from Bligh and Dyer [55]. Dried lipid extracts were dissolved in 200 μL of methanol. Then, 20 μL of the lipid extract in methanol was loaded into 96-well plates and diluted with 20 μL of 20 mM ammonium acetate in methanol. Lipid infusion and ionization were conducted using Nano-ESI chips with the TriVersa NanoMate operated by the ChipSoft Software (Advion) under the following settings: sample infusion, 14 μL; volume of air-to-aspirate after sample, 1.0 μL; air gap before chip, enabled; aspiration delay, 0 s; pre-piercing, with mandrel; spray sensing, enabled; cooling temperature, 14 °C; gas pressure, 0.5 psi; ionization voltage, 1.4 kV; and vent space, enabled. Prewetting was performed once. 

Mass spectrometric analysis was performed using the QTRAP 6500 (SCIEX) operated by Analyst 1.6.3. The following instrument-dependent configurations were used: curtain gas at 50 psi; CAD gas at medium mode; and interface heater temperature at 100 °C. Detection of triglyceride species was conducted by scanning for the neutral losses of ammonium adducts of distinct fatty acids: *m*/*z* 273 (16:0), 295 (18:3), 297 (18:2), 299 (18:1), 301 (18:0), 321 (20:4) and 345 (22:6). A mass range of m/z 750−1100 Da was scanned using a collision energy of 40 eV. Cholesteryl ester species were detected by scanning for precursors of m/z 369 within a mass range of *m*/*z* 580–790 Da using a collision energy of 15 eV. All scans were conducted in the positive ion mode at a scan rate of 200 D/s with a declustering potential of 100 V, a potential of 10 V and a cell exit potential of 14 V(15). Mass spectra were processed by the LipidView Software version 1.2 (SCIEX) for identification and quantification of lipids. Endogenous lipid species were quantified by normalizing their peak areas to those of the internal standards. The calculated amounts of triglycerides and cholesteryl esters were normalized to the plasma volume and meibum weight used for the analysis, respectively.

#### 4.3.2. Analysis of Ceramides and Sphingomyelins

Ceramide and sphingomyelin species were quantified by liquid chromatography coupled with electrospray ionization tandem mass spectrometry (LC-ESI-MS/MS). To 50 µL of plasma, 50 µL of Milli-Q water and 750 µL of methanol/chloroform 2:1 (*v*/*v*) were added. Dry meibum was resolved in 100 µL of Milli-Q water and 750 µL of methanol/chloroform 2:1 (*v*/*v*). Lipid extraction and LC-ESI-MS/MS analysis were performed as previously described [56,57]. The calculated amounts of ceramides and sphingomyelins were normalized to the plasma volume and meibum weight used for the analysis, respectively.

### 4.4. Nomenclature and Presentation of Lipid Quantities

The following lipid names and abbreviations are used: cholesteryl ester (CE), triglycerides (TG), ceramides (Cer) and sphingomyelins (SM). Lipid species were annotated according to their molecular composition as follows: [lipid class]-[sum of carbon atoms]:[sum of double bonds in the fatty acid moiety or degree of unsaturation in the respective acyl chains], e.g., [CE-14:0]. Lipids species with no double bonds in their fatty acids moieties are termed saturated, those with a single double bond are termed monounsaturated fatty acids (MUFAs) and those with two or more double bonds in their fatty acids are termed polyunsaturated fatty acids (PUFAs). Quantitative lipid data were obtained initially as µmol/mL and nmol/mg per lipid class for plasma and meibum, respectively. Relative abundances (distributions) of the lipid species were calculated and presented as mol% of all species of the given class set to 100% [27].

### 4.5. Image and Statistical Analyses

Meibography images were analyzed using Image J (NIH, Bethesda, MD, USA); the details of this are described in Appendix A. Data were analyzed using GraphPad Prism 6 (GraphPad Software, La Jolla, CA, USA) and R (version 3.02 for Windows). Normality of data was assessed quantitatively using the Shapiro–Wilk test, and Levene’s test was used to assess the equality of variances. In most cases, the data passed normality, and binary logarithmic transformation was performed in the few cases where data were not normally distributed. Data were obtained from n = 8 animals per group. Sample size estimate was based on a previously published meibomian gland morphometric analysis [34]; using a conservative assumption of a standard deviation of 0.26, it was determined that to obtain a power of 0.8, approximately n = 5 mice /group were needed, but three extra mice were added to each group to account for possible mortality and technical issues. Measurement of TGs and CEs was performed only once per sample, whereas for Cer and SMs, two technical replicates were performed per sample. Unpaired t-tests were performed and Holm–Sidak’s correction was applied for multiple comparisons of lipid species levels between the ND and HFD groups. Data are presented as mean and standard error of the mean (SEM) or standard deviation (SD), as appropriate. Pearson’s correlation and principal component analyses were also performed using a custom R code. A *p*-value ≤ 0.05 was considered significant.

## Figures and Tables

**Table 1:** List of various fatty acid moieties of the lipid species detected in plasma and meibum.

Common Name	Chemical Formula	* C:D	Known * mp (°C)	Plasma	Meibum
Myristic acid	C_14_H_28_O_2_	14:0	53.9	✓	✓
Pentadecylic acid	C_15_H_30_O_2_	15:0	51–53		✓
Palmitic acid	C_16_H_32_O_2_	16:0	61.8	✓	✓
Margaric acid	C_17_H_34_O_2_	17:0	61.3		✓
Stearic acid	C_18_H_36_O_2_	18:0	69.6	✓	✓
Nonadecylic	C_19_H_38_O_2_	19:0	68.0–70.0		✓
Arachidic acid	C_20_H_40_O_2_	20:0	77.0	✓	✓
Heneicosylic acid	C_21_H_42_O_2_	21:0	74.0–75.0		✓
Behenic acid	C_22_H_44_O_2_	22:0	81.5	✓	✓
Tricosylic acid	C_23_H_46_O_2_	23:0	77.0–79.0		✓
Lignoceric acid	C_24_H_48_O_2_	24:0	88.0	✓	✓
Pentacosylic acid	C_25_H_50_O_2_	25:0	* n/a		✓
*Cerotic acid*	C_26_H_52_O_2_	26:0	88.5	✓	✓
Myristoleic acid	C_14_H_26_O_2_	14:1	−4.0		✓
Palmitoleic acid	C_16_H_30_O_2_	16:1	−0.5–−0.1	✓	✓
Oleic acid	C_18_H_34_O_2_	18:1	12.0	✓	✓
Paullinic acid	C_20_H_38_O_2_	20:1	13.4		✓
Erucic acid	C_22_H_42_O_2_	22:1	33.8	✓	✓
Nervonic acid	C_24_H_46_O_2_	24:1	39.0	✓	✓
Ximenic acid	C_26_H_50_O_2_	26:1	50.5–50.9	✓	✓
Linoleic acid	C_18_H_32_O_2_	18:2	−5.0	✓	✓
α- Linoleic acid	C_18_H_30_O_2_	18:3	−11.0	✓	✓
Eicosatrienoic acid	C_20_H_34_O_2_	20:3	n/a	✓	✓
Arachidonic acid	C_20_H_32_O_2_	20:4	−49.5	✓	✓
Eicosapentanoic acid	C_20_H_30_O_2_	20:5	−54.0	✓	
Docosapentanoic acid	C_22_H_34_O_2_	22:5	−78.0	✓	
Docosahexaenoic acid	C_22_H_32_O_2_	22:6	−44.0	✓	✓

* C:D = total number of carbon atoms: total number of double bonds, * mp = melting point in degrees Celsius, * n/a = not available. Rows with dark background indicate a discrepancy between plasma and meibum.

**Figure A1:**
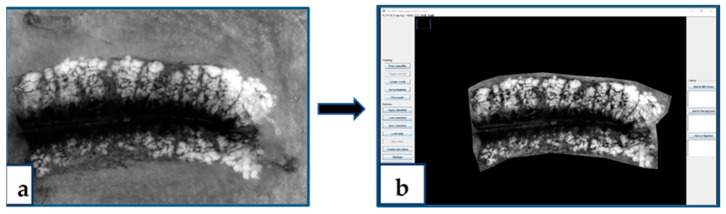
Raw meibograph acquired with the STEMI microscope (**a**) and loaded into image J; then, excess eyelid area was cropped after the CLAHE filter was applied (**b**).

**Figure A2:**
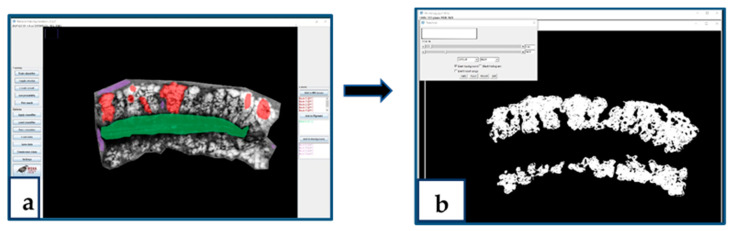
Classifiers are applied and segmentation ran (**a**) and the first probability map with a threshold for further quantitative analysis (**b**).

**Figure A3:**
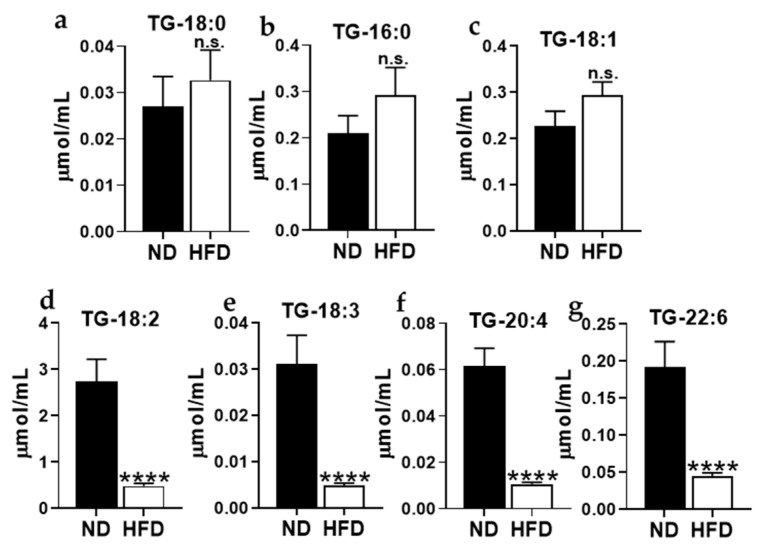
Total levels of plasma (**a**) TG-16:0, (**b**) TG-18:0, (**c**) TG-18:1, (**d**) TG-18:2, (**e**) TG-18:3, (**f**) TG-20:4 and (**g**) TG-22:6. Data are expressed as means ± SEM, **** *p* < 0.0001, n.s = not significant. Asterisks indicate significant difference compared to the matched ND value.

**Figure A4:**
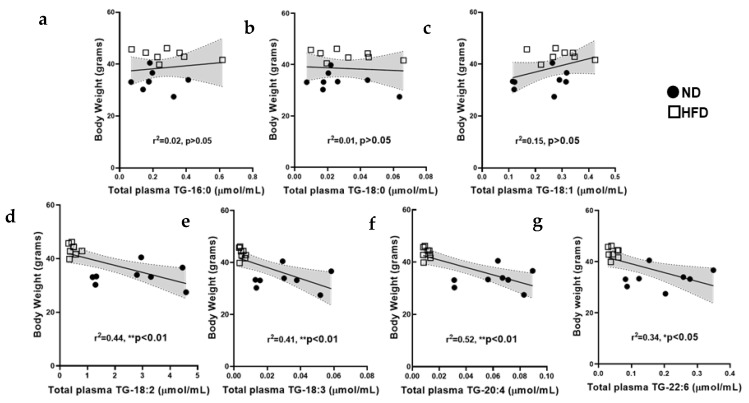
Correlation between mouse body weight and total plasma (**a**) TG-16:0, (**b**) TG-18:0, (**c**) TG-18:1, (**d**) TG-18:2 (**e**) TG-18:3 (**f**) TG- 120:4 and (**g**) TG-22:6. The grey-shaded regions represent the 95% confidence interval. Asterisks indicate a significance difference compared to the matched ND value.

## Abbreviations

eAT	Epididymal adipose tissue
CE	Cholesteryl ester
Cer	Ceramide
HFD	High-fat diet
MGD	Meibomian gland dysfunction
MUFAs	Monounsaturated fatty acids
ND	Normal diet
PUFAs	Polyunsaturated fatty acids
SM	Sphingomyelins
TG	Triglcyerides

## Appendix A. Analysis of Meibographs

Meibographs were acquired with a Zeiss Stemi 305 microscope (Carl Zeiss Microscopy, White Plains, NY, USA). This is a Greenough-type stereo-microscope with integrated LED illumination and an attached camera system with black and white and color imaging modes. The stereo-microscope comes with a high-speed serial bus converter that is connected to a computer for image acquisition, storage and processing using the custom Zen Lite software.

Using custom settings, we acquired 2560 × 1920 pixels meibographs. The raw image files were exported into image J for analysis. We employed a semi-automated threshold intensity segmentation approach. Images were opened in Image J and the CLAHE filter was applied (*Process → CLAHE→fast rendering option*) to enhance image contrast. After this, the polygon selection function was used to define boundaries around the meibomian glands and the excess eyelid area without gland was cropped out using the *Edit → Clear Outside* function (Figure A1).

In the next step, we applied a custom WEKA trainable segmentation with three defined classifiers [58]:

1. Meibomian gland tissue.
2. Pigmentation—little darker spots sometimes on the glandular tissue and very dense along the lid margins.
3. Background—includes areas without glands.

After defining and applying the classifiers, the *“Create Result”* tab was clicked. The WEKA segmentation should be completed in about 1 min or less depending on computer system specifications.

After this step, probability maps were generated and the first probability map, which is essentially a black and white image, was selected for thresholding (*Image → Adjust → Threshold*) to define the glands (Figure A2). All meibographs were calibrated using a 500-μm scale bar auto-generated by the Zeiss Stemi 305 microscope. Glandular area (using the free hand selection tool) and lengths (using the freehand line selection tool) were then performed routinely using the *Analyze → Measure* function.

## Appendix B. Principal Component Analysis Using Custom R Code

Principal component analysis (PCA) is a data dimension reduction method used to extract important information from multivariate data and to present this information as a set of few new variables called principal components [59]. These new variables correspond to a linear combination of the originals. Since the information in a given data set that corresponds to the total variation, PCA, therefore, enables us to identify directions (principal components) along which data variation is maximal [59]. In using a custom R code and relative abundance data of plasma and meibum CE, TG, Cer and SM lipid species from ND and HFD mice, we generated variable correlation plots that show the direction and magnitude of the variation per the various lipid species. Visit Statistical Tools for High-throughput Data Analysis for useful resources.

## Appendix C. Total Fasting Levels of Various Plasma Triglycerides Species

Triglycerides (TG) contain three fatty acids; therefore, total TG levels were derived differently compared to CE, Cer and SM lipid classes. For TG, total levels were derived by adding the various TG species separate from each other. Figure A3 are plots for the total levels of the various TG species.
